# Editorial: Advanced Cardiovascular Imaging in Diabetes

**DOI:** 10.3389/fendo.2022.848975

**Published:** 2022-03-18

**Authors:** Fritz Schick, Rasmus Sejersten Ripa, Tine Willum Hansen, Bernt Johan von Scholten

**Affiliations:** ^1^ Section on Experimental Radiology, Department of Diagnostic and Interventional Radiology, Tübingen University Hospital, Tübingen, Germany; ^2^ Department of Clinical Physiology, Nuclear Medicine and PET, Rigshospitalet, Copenhagen, Denmark; ^3^ Complications Research, Steno Diabetes Center Copenhagen (SDCC), Gentofte, Denmark; ^4^ Global Chief Medical Office, Novo Nordisk, Copenhagen, Denmark

**Keywords:** diabetes, cardiovascular disease, imaging, atherosclerosis, type 2 diabetes, inflammation

This Research Topic asked the question - which insights have we learned on cardiovascular disease in diabetes and what more can we gain from advanced cardiovascular imaging in the future?

The connection between diabetes and cardiovascular disease is clear and individuals with diabetes are at least two times more likely to develop cardiovascular disease, which is the leading cause of morbidity and mortality in diabetes (1). Diabetes itself confers an independent risk of cardiovascular disease, but conditions that often coexist with diabetes such as hypertension and dyslipidemia are also important risk factors (2). Recent work suggests that imaging techniques can detect significant changes in diabetic cardiomyopathy long before symptoms appear (3).

The earliest non-invasive technique for visualising the contour of the heart is projection radiography, which has been available for more than 120 years. This technique can help to clarify relatively simple anatomical questions but is still used today as an initial examination for many clinical indications. Computed tomography (CT), introduced in the 1970s, enables the acquisition of spatially highly resolved three-dimensional anatomical data sets. With modern CT systems using multi-slice detectors and two X-ray tubes, data acquisition times of about 100 ms are possible for extended volume data sets with submillimetre resolution (4). Ultrasound (US) methods do not require radiation exposure, are relatively inexpensive, and at the same time allow a rapid temporal repetition of individual measurements. For studies of blood flow in vessels and in the heart chambers, ultrasound imaging methods can be combined with Doppler-based velocimetry and recorded together with wall motion (5). Since the 1980s, magnetic resonance imaging (MRI) has developed rapidly and is now a very powerful modality for high spatial resolution morphological and functional studies of the heart and peripheral vessels (6). The use of specific radioactive tracers within nuclear medicine can show physiological properties such as ischaemia or inflammatory activity (7). In contrast to traditional scintigraphy with gamma cameras, positron emission tomography (PET) has a very high sensitivity for recording 3D data sets even with extremely low tracer concentrations (8).

In recent years, multimodal and hybrid imaging techniques have been improved and developed into the new frontier of medical molecular imaging with the possibility to overcome restrictions of single modalities and to acquire non-invasive physiological and pathophysiological measurements within an accurate anatomical framework. These advanced imaging methods can directly assess both function and morphology of the heart and vessels and have the capacity to redefine our understanding of cardiovascular disease, to improve the pathological understanding, to refine risk stratification and to select persons for targeted therapies. Moreover, studies using advanced imaging has the potential to improve our understanding of the cardiovascular mode of action of novel therapies. All these aspects are covered in the topic with up-to-date articles on studies in which modern imaging methods were used to elucidate relationships between diabetic conditions and cardiovascular effects.

Two papers were within the frame of novel methods aimed for better risk stratification. The work of Chen et al. evaluates the utility of color M-mode derived aortic propagation velocity (APV) to recognizing alterations in the fetal vascular endothelial function. The APV was lower in fetuses from women with gestational diabetes and correlated with fetal endothelial function. Thus, this novel metric may represent a promising non-invasive and simple method to assess endothelial function in fetuses. This might identify initial alterations of vascular function, even in women with well-controlled gestational diabetes and the measurement of APV might be used in fetuses referred for a fetal echocardiography, to improve cardiovascular risk estimation, for better selection of high-risk individuals and for corresponding preventive interventions. Along similar lines, the work of Shabani et al. tested the hypothesis that soft tissue calcification could be triggered by prolonged hyperglycaemia. For this purpose, costal cartilage calcification in non-contrast CT images of 2305 individuals in the Multi-Ethnic Study of Atherosclerosis (MESA) was correlated with prior fasting blood glucose levels in a sex-dependent manner. The hypothesis was only confirmed in women regardless of coronary artery calcium score. Further validation of the potential role for APV and costal cartilage calcification for risk stratification is needed, including studies in larger prospective cohorts with follow-up data.

The article by Mavrogeni et al. provides an overview of common MRI questions regarding myocardium and vascular system in diabetic patients. Examples and the state-of-the-art MRI methods for anatomical and functional investigations in ischaemia, infarction and structural changes of the myocardium with fatty degeneration and fibrosis are presented. To further build upon this Research Topic, Seetharam et al. discuss the potential role of machine learning in the field of advanced cardiovascular imaging. The authors conclude that machine learning-driven algorithms will be inevitable, as they have the potential to significantly augment the workflow of the very large information content of the recorded data.

Petersen et al. ask the important question on how to facilitate the use of non-invasive cardiovascular imaging in clinical practice. The study compares the activity of non-invasive cardiovascular imaging in the US (Medicare fee-for-service, 2011–2015) and England (National Health Service, 2012–2016). The imaging activity was three-times higher in the US compared to England. This highlights the importance of reimbursement as a main driver for the clinical application, central knowledge for the policy makers.

One paper applies advanced imaging to improve our understanding of the cardiovascular mode of action of a novel therapy. In this Research Topic, Jensen et al. report on a patient study with 26 weeks of therapy with the glucagon-like peptide-1 receptor agonist liraglutide to investigate if this substance has a direct anti-inflammatory effect in the coronary arteries. The effect of this medication on the uptake of the novel radiotracer [^64^Cu]Cu-dotatate, which binds to somatostatin receptors, was investigated in the coronary arteries using hybrid PET/CT imaging. Liraglutide treatment caused a significant reduction in [^64^Cu]Cu-DOTATATE uptake in the coronary arteries whereas this was not seen in the placebo treated group. The difference in change in uptake between the two groups did however only reach borderline statistical significance.

The paper from Laursen et al. aims to enhance our understanding of the development of cardiovascular disease using advanced imaging. The study investigates the relationship between baseline cardiovascular autonomic function and diabetic renal and myocardial sequelae over six years in 24 persons with type 2 diabetes and a group of healthy controls. The coronary function was measured using cardiac ^82^Rb PET/CT. Cardiac autonomic dysfunction at baseline was found associated with a larger decline in kidney function during six years of follow up, but not with changes in coronary function or atherosclerotic burden. The investigated population was however small and a considerable risk of selection- and survival bias was present. Thus, the results should be interpreted as exploratory and further studies in larger populations are warranted.

## Author Contributions

All authors listed have made a substantial, direct, and intellectual contribution to the work and approved it for publication.

## Conflict of Interest

BJvS is employed at the company Novo Nordisk.

The remaining authors declare that the research was conducted in the absence of any commercial or financial relationships that could be construed as a potential conflict of interest.

## Publisher’s Note

All claims expressed in this article are solely those of the authors and do not necessarily represent those of their affiliated organizations, or those of the publisher, the editors and the reviewers. Any product that may be evaluated in this article, or claim that may be made by its manufacturer, is not guaranteed or endorsed by the publisher.
